# Antioxidant supplementation increases retinal responses and decreases refractive error changes in dogs

**DOI:** 10.1017/jns.2016.5

**Published:** 2016-05-10

**Authors:** Wei Wang, Jerome Hernandez, Cecil Moore, Janet Jackson, Kristina Narfström

**Affiliations:** 1 Nestlé Research Center, St Louis, MO, USA; 2 Nestlé Purina Research, St Joseph, MO, USA; 3 Department of Veterinary Medicine and Surgery College of Veterinary Medicine, University of Missouri, Columbia, MO, USA; 4 Department of Ophthalmology, Mason Eye Institute, University of Missouri, Columbia, MO, USA

**Keywords:** Antioxidants, Dogs, Canine nutrition, Electroretinography, Retina, Refractive error, Visual acuity, ERG, electroretinography, Pc, photopic single-flash, Pfl, photopic flicker, PRCD, progressive rod–cone degeneration, Sh, scotopic high-intensity, Ssd, scotopic standard intensity

## Abstract

The objective of the study was to examine whether a nutritional antioxidant supplementation could improve visual function in healthy dogs as measured by electroretinography (ERG) and autorefraction. A total of twelve Beagles, 6 to 8 years of age, with normal eyes upon indirect ophthalmoscopy and slit lamp biomicroscopy, were age and sex matched and randomly assigned to receive a feeding regimen for 6 months with or without a daily antioxidant supplementation. Portable, mini-Ganzfeld ERG and a Welch Allyn hand-held autorefractor were used to test retinal response and refractive error in the dogs at baseline and at the end of the supplementation period. All ERG a-wave amplitudes obtained were increased in the treatment group compared with those of dogs in the control group, with significant improvements in the scotopic high and photopic single flash cone ERG responses (*P* < 0·05 for both). For the b-wave amplitudes, all responses were similarly increased, with significant improvements in responses for the scotopic high light intensity stimulation (*P* < 0·05), and for photopic single flash cone and 30 Hz flicker (*P* < 0·01 for both) recordings. Change in refractive error was significantly less in the treatment group compared with that of the control group during the 6-month study (*P* < 0·05). Compared with the control group, the antioxidant-supplemented group showed improvement to varying degrees for retinal function and significantly less decline in refractive error. Dogs, like humans, experience retinal and lens functional decline with age. Antioxidant supplementation as demonstrated may be beneficial and effective in the long-term preservation and improvement of various functions of the canine eye.

Eyesight is one of the key senses for acquiring information from the outside world. Dogs, like humans, experience eye changes with ageing. Retinal degeneration and cloudy lens (nuclear sclerosis) are common forms of eye problems that result in a decline of visual function in dogs.

A healthy retina is a critical component for overall visual function in an animal. Electroretinography (ERG) has been used as an objective, well-established method to evaluate retinal function in dogs^(^^1^^)^. ERG responses may vary among different breeds and ERG a- and b-wave amplitudes appear to decrease with ageing^(^^2^^)^.

A healthy or clear lens is another important factor in overall visual function in an animal. Development of cloudy lenses in older dogs, referred to as nuclear sclerosis, occurs with the ageing process^(^^3^^)^. The cloudy lens of older dogs is readily visible to the naked eye as an observed hazy or bluish appearance within the pupil space and often is viewed by owners as suspected cataract formation. Nuclear sclerosis was the second most commonly diagnosed condition recorded for geriatric dogs greater than 10 years of age in a 2011 study conducted by a privately owned US pet hospital chain^(^^4^^)^.

In veterinary ophthalmology, nuclear sclerosis is not generally believed to significantly affect vision in dogs, except in unusually dense or advanced cases. However, the clinical distinction between advanced nuclear sclerosis and early nuclear senile cataract in dogs is often indistinct^(^^5^^)^. Similar nuclear sclerosis changes in humans are considered a type of cataract which may be associated with lens nuclear brunescence and myoptic shift in older humans and is referred to as nuclear cataract or senile cataract^(^^6^^,^^7^^)^.

Whether age-related lens changes such as nuclear sclerosis might result in any vision impairment in dogs is hard to study since dogs cannot communicate like humans do, and most human vision tests rely on the human's feedback for evaluations. Some objective visual function measurement techniques do exist, however. One such method uses a 5-s autorefractor for testing refractive errors in infants and toddlers who cannot yet communicate well or at all. The portable handheld autorefractors have lights and sounds that engage test subjects’ attention, with minimal cooperation required^(^^8^^,^^9^^)^. We and others have previously tested and validated this method for visual function assessment in dogs and found it a repeatable method to use in dogs^(^^10^^,^^11^^)^.

Previous studies to examine the impact of nutrition on visual function in dogs had focused on fish oil. Fish oil supplementation was shown to increase the PUFA levels in normal dogs, but was unable to correct the lipid abnormalities (low fatty acid levels) in dogs with progressive rod–cone degeneration (PRCD). ERG responses were not improved in either the dogs with PRCD or normal control dogs even after 21 weeks of supplementation^(^^12^^)^. In another study, ERG responses were significantly improved in 12-week-old puppies who received high amounts of fish oil throughout gestation, lactation and weaning^(^^13^^)^.

The literature for human eye health has shown extensively that dietary lutein, zeaxanthin and β-carotene, and other major antioxidants, such as vitamins C and E, are inversely associated with risks of eye diseases, such as age-related macular degeneration and cataracts^(^^14^^–^^21^^)^. Carotenoids and other antioxidants may be beneficial because they absorb light (leading to less light damage), and act as antioxidants that protect the retina and lens from oxidative damage. Combinations of various antioxidants have been shown to improve central visual function in human subjects with macular degeneration^(^^16^^,^^18^^–^^21^^)^. These nutritional factors may have beneficial effects on dogs’ eyes and visual functions.

To our knowledge, there have been no prior reports on the effects of nutritional factors, such as lutein, zeaxanthin and other antioxidants on visual function measurements in dogs. The objective of the present study was to examine whether a combination of antioxidants, known to have eye health benefits in humans, can improve retinal function and/or decrease visual impairment associated with lens ageing in dogs.

## Materials and methods

### Animals

A total of twelve adult Beagles (eight males and four females) between the ages of 6 and 8 years, with body condition scores between 4 and 5 (scale of 1–9), were recruited for the study. Two of the four female dogs were spayed and two were intact; neither was pregnant or lactating during the study period. Only dogs with a normal appearing fundus and without abnormalities of the eyes were included in the trial. Prior to the study and at the end of the study, an ophthalmic evaluation was performed by a veterinary ophthalmologist (K. N.) via slit lamp biomicroscopy and indirect ophthalmoscopy. The dogs’ pupils were dilated with short-acting mydriatic eye drops, 1 % tropicamide (Mydriacyl^®^), at least 15 min prior to the examinations.

Throughout the study, the dogs were housed either individually or in pairs, with continuous free access to be indoor or outdoor, and were fed individually to maintain body weight. Body weights were monitored and amounts of dry food adjusted so that body weight did not vary more than 10 % from initial body weight. All dogs were provided with opportunities for outdoor exercise and social interactions. Dogs were provided with water *ad libitum* except when fasted prior to anaesthesia. This study protocol was reviewed and approved by the Nestlé Purina Animal Care and Use Committee.

The dogs were divided into two groups, matched for age, body weight and sex. The groups were randomised to receive either antioxidant supplements, or control diets only, during the treatment period.

### Food and supplements

All dogs were fed a nutritionally complete and balanced dry dog food (see Table 1: dry food) twice daily throughout the 1-month baseline and 6-month study periods. The dogs received no vitamin C from the control diet. During the study period, dogs in the treatment group received a daily supplement of antioxidant blend (lutein 20 mg, zeaxanthin 5 mg, β-carotene 20 mg, astaxanthin 5 mg, vitamin C 180 mg, and vitamin E 336 mg per d, with cellulose as the blending medium) topped with a small quantity of canned food (see Table 1: wet food). Prior to each morning meal, dogs in the treatment group received a 2·75 oz (approximately 97 kcal) (78 g; approximately 406 kJ) portion of canned dog food to which the antioxidant blend was added. Control dogs received the canned portion without added antioxidants. Dogs were monitored daily and were weighed weekly throughout the baseline and study periods.

**Table 1.** Nutrient composition of dry and wet foods*

Nutrient composition	Dry food content	Wet food content
Moisture (%)	8·3	75·0
Fat (%)	16·4	9·6
Protein (%)	27·8	11·0
Carbohydrate (%)	39·1	1·8
Ash (%)	7·0	2·7
Fibre (%)	1·4	0·1
Energy		
kcal/kg diet	3758	97
kJ/kg diet	15723	406
Vitamin C (mg/kg diet)	0	0
Vitamin E (mg/kg diet)	39	0

* The dry food base diet used in the study is a nutritionally complete and balanced diet meeting AAFCO (Association of American Feed Control Officials) requirements for vitamins C and E. The wet food is a commercial canned dog food.

### Vision tests

ERG was used to assess the retinal response of the dogs’ retinal function; a hand-held auto-refractor was used to assess the refractive error changes (a measure of visual impairment) of the dogs’ lens at the baseline and the end of the 6-month supplementation.

### Electroretinography

ERG was performed by a veterinary ophthalmologist (K. N.) using portable, mini-Ganzfeld ERG equipment (HMsERG model 1000; RetVet Corp.) as previously described^(^^22^^)^, with an automated and standardised canine ERG protocol. The examiner (K. N.) was masked to the dogs’ treatment group.

Individual ERG were recorded for each dog at the end of the baseline period and again at the end of the 6-month study period. Each ERG session consisted of scotopic and photopic ERG in accordance with the Dog Diagnostic Protocol, recommended by the European College of Veterinary Ophthalmologists, primarily for evaluation of rod and cone function^(^^1^^)^. This protocol was pre-programmed on the ERG unit and was executed automatically on initiation of the ERG session by the examiner. During 20 min of dark adaptation, scotopic low-intensity light stimuli responses (S) were elicited every 4 min (S1–S5) at 0·01 cd·s/m^2^; averaged responses to ten flashes, given at 2-s intervals (0·5 Hz), were recorded for each time point in order to evaluate pure rod function. The light stimulus intensity was then increased to 3 cd·s/m^2^ and the averaged responses to four flashes at 10-s intervals were recorded (Ssd). Thereafter, scotopic high-intensity (Sh) responses were elicited, using 10 cd·s/m^2^; averaged responses to four flashes administered at 20-s intervals were recorded. The latter two recordings depict responses from a mixture of rods and cones. After 10 min of light adaptation, with a background luminance of 30 cd·s/m^2^, photopic single-flash (Pc) responses were recorded using 3 cd·s/m^2^ of flash stimulus, averaging thirty-two flashes at an interval of 0·5 s, followed by evaluation of 30-Hz photopic flicker (Pfl) for a total of 4·1 s at the same light intensity stimulation. The latter two recordings were performed to evaluate cone and cone inner retina pathways, respectively.

Data were collected automatically on the compact flash card of the ERG unit, transferred to a computer, printed and stored for further analysis. ERG tracings were evaluated, and the amplitudes and implicit times for the a- and b-waves were measured as previously described^(^^1^^)^.

### Hand-held autorefractor

Spherical equivalent refractive error was measured by handheld autorefractor (Welch Allyn SureSight) on separate days from the ERG measurement. The dogs’ eyes were not dilated for autorefractor tests. Autorefractor measurements were done under indirect lighting conditions with five measurements per eye. The indirect lighting condition was set with indoor light from an adjacent room coming through an open door into a dark room with dogs facing the incoming light (illumination about 125 lux). Refractive error was calculated as sphere +0·5 cylinder.

We had previously validated the use of this autorefractor technique in a group of dogs 1–14 years of age under set light conditions, three times over a 6-week time period, and found that the measurements were repeatable, and the refractive errors was significantly associated with the age of the dogs^(^^10^^)^.

### Statistics

The data were analysed using non-parametric Wilcoxon–Mann–Whitney test (PROC NPAR1WAY in SAS 9.2), comparing the differences between treatment and control groups for the change from initial test values (post–pre test). Results are expressed as means with their standard errors. Statistical significant difference was defined at *P* < 0·05 (SAS 9.2).

## Results

All the dogs completed the study in good health. The body weight (see Table 2) of all the dogs was maintained throughout the 7 months of this study.

**Table 2.** Characteristics of the dogs (Mean values with their standard errors, or number)

	Age (years)		Sex (*n*)		Body weight (kg)	
Group	Mean	se	Female	Male	Mean	se
Control	6·76	0·29	2	4	10·81	0·36
Treatment	6·82	0·34	2	4	10·92	0·76

The baseline values for all ERG parameters did not differ for the treatment groups. Despite a large individual variation in ERG responses throughout the study, which resulted in the large standard error of the measured parameters, several parameters were significantly different for the treatment and control groups (see Table 3). There were statistically significant differences observed in the scotopic ERG parameters (Ssd and Sh, measures for rod function under standard and high-intensity light stimuli under dark adapted conditions) and in the photopic ERG parameters (Pc, a measure for cone function, and Pfl, a measure for cone and rod function, both under light-adapted conditions) of the treatment group and the control group. The strong effect sizes for these parameters are reported with *P* values in Table 3.

**Table 3.** Electroretinography (ERG) outcome measurements in two study groups before (pre-test) and after treatment (post-test)† (Mean values with their standard errors)

Group…		Control				Treatment				
		Pre-test		Post-test		Pre-test		Post-test		
		Mean	se	Mean	se	Mean	se	Mean	se	Effect size
a-Wave amplitude (μV)	Ssd	104·08	8·64	115·78	9·13	112·78	8·13	150·55	21·30	0·91
	Sh	151·92	9·41	147·08	10·43	149·35	12·05	183·37	19·33	1·39*
	Pc	10·88	1·74	10·25	1·77	10·32	1·34	13·12	2·87	1·99**
a-Wave implicit time (ms)	Ssd	15·27	0·64	15·53	0·53	15·58	0·40	14·00	0·62	1·43*
	Sh	13·40	0·68	14·42	1·29	14·23	1·08	12·98	0·73	1·12
	Pc	12·02	0·34	12·30	0·62	12·50	0·38	10·18	2·09	0·33
b-Wave amplitude (μV)	Ssd	167·38	10·92	167·68	13·75	168·60	13·30	214·87	26·75	1·38*
	Sh	198·32	11·71	185·57	16·48	192·55	14·14	236·77	31·18	1·61*
	Pc	43·90	4·04	38·60	5·06	45·77	6·30	52·44	6·24	2·73**
	Pfl	46·05	7·94	35·80	6·96	37·77	4·70	54·55	9·28	1·89**
b-Wave implicit time (ms)	Ssd	33·18	0·89	35·35	0·81	33·50	0·76	35·55	0·57	0·04
	Sh	33·92	1·24	36·28	0·87	37·23	0·98	37·62	0·49	0·59
	Pc	25·08	1·04	25·93	1·46	25·65	0·28	21·53	3·59	0·74
	Pfl	22·78	0·19	23·42	0·70	23·10	0·13	23·17	0·20	0·59

Ssd, ERG response from scotopic standard-intensity light stimulation; Sh, response from scotopic high-intensity light stimulation; Pc, photopic cone response; Pfl, photopic flicker (30 Hz) response.

Statistical analyses of treatment × time interaction: * *P* < 0·05, ** *P* < 0·01.

† See text for the specific light intensities used.

**Fig. 1.**
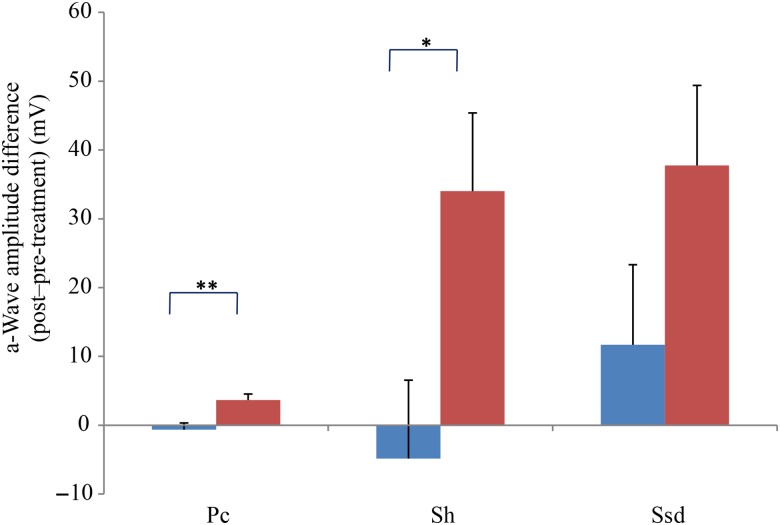
a-Wave amplitude difference from post- to pre-treatment tests in treatment (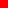) *v.* control (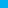) groups. Ssd and Sh: with higher light intensity stimulation (Ssd and Sh: 3 and 10 cd·s/m^2^, respectively) in the dark-adapted state, a- and b-wave responses are obtained, demonstrating mixed rod and cone function. Pc: rods are desensitised using 30 cd·s/m^2^ of background light for 10 min, after which cones are stimulated using 3 cd·s/m^2^ in the light-adapted state. Values are means, with standard errors represented by vertical bars. Significant treatment × time interaction: * *P* < 0·05, ** *P* < 0·01. Ssd, electroretinography response from scotopic standard-intensity light stimulation; Sh, response from scotopic high-intensity light stimulation; Pc, photopic cone response.

**Fig. 2.**
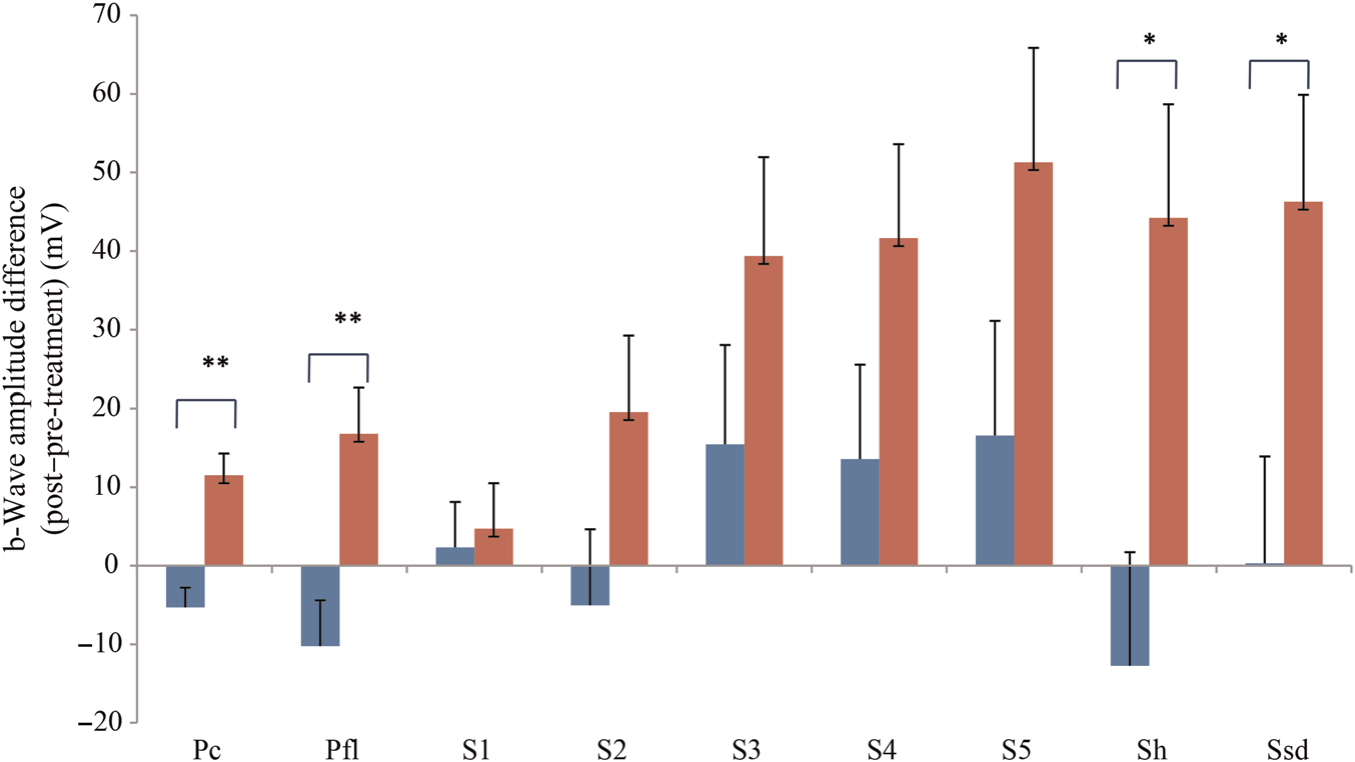
b-Wave amplitude difference from post- to pre-treatment tests in treatment (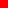) *v.* control (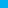) groups. Ssd and Sh: with higher light intensity stimulation (Ssd and Sh: 3 and 10 cd·s/m^2^, respectively) in the dark-adapted state, a- and b-wave responses are obtained, demonstrating mixed rod and cone function. Pc and Pfl: rods are desensitised using 30 cd·s/m^2^ of background light for 10 min, after which cones are stimulated using 3 cd·s/m^2^ in the light-adapted state. Pc and Pfl: single flash and 30 Hz flicker. S (S1–S5): using low intensity of light stimulation (0·01 cd·s/m^2^) in the dark-adapted state every 4 min up to 20 min of dark adaptation; using this low light intensity only the b-wave is obtained, corresponding to activity from the rod photoreceptors. Values are means, with standard errors represented by vertical bars. Significant treatment × time interaction: * *P* < 0·05, ** *P* < 0·01. Ssd, electroretinography response from scotopic standard-intensity light stimulation; Sh, response from scotopic high-intensity light stimulation; Pc, photopic cone response; Pfl, photopic flicker (30 Hz) response.

**Fig. 3.**
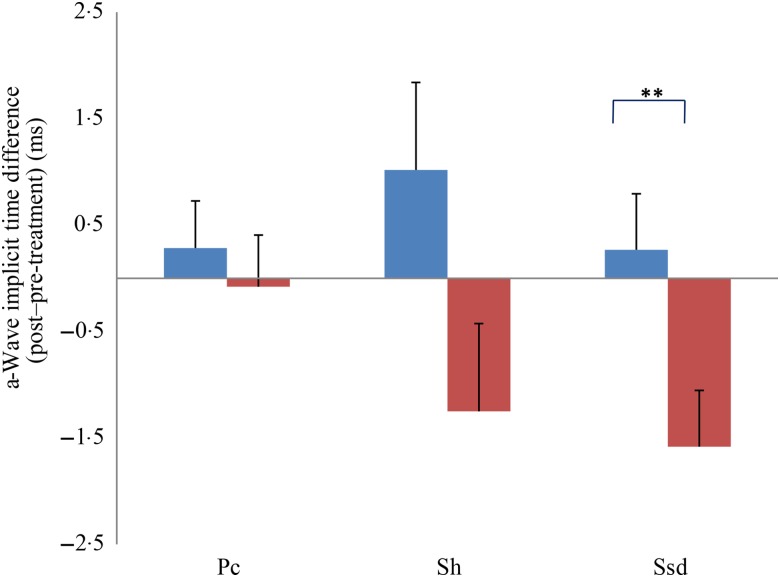
a-Wave implicit time difference from post- to pre-treatment tests in treatment (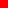) *v.* control (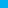) groups. Ssd and Sh: with higher light intensity stimulation (Ssd and Sh: 3 and 10 cd·s/m^2^, respectively) in the dark-adapted state, a- and b-wave responses are obtained, demonstrating mixed rod and cone function. Pc: rods are desensitised using 30 cd·s/m^2^ of background light for 10 min, after which cones are stimulated using 3 cd·s/m^2^ in the light-adapted state. Values are means, with standard errors represented by vertical bars. Significant treatment × time interaction: ** *P* < 0·01. Ssd, electroretinography response from scotopic standard-intensity light stimulation; Sh, response from scotopic high-intensity light stimulation; Pc, photopic cone response.

**Fig. 4.**
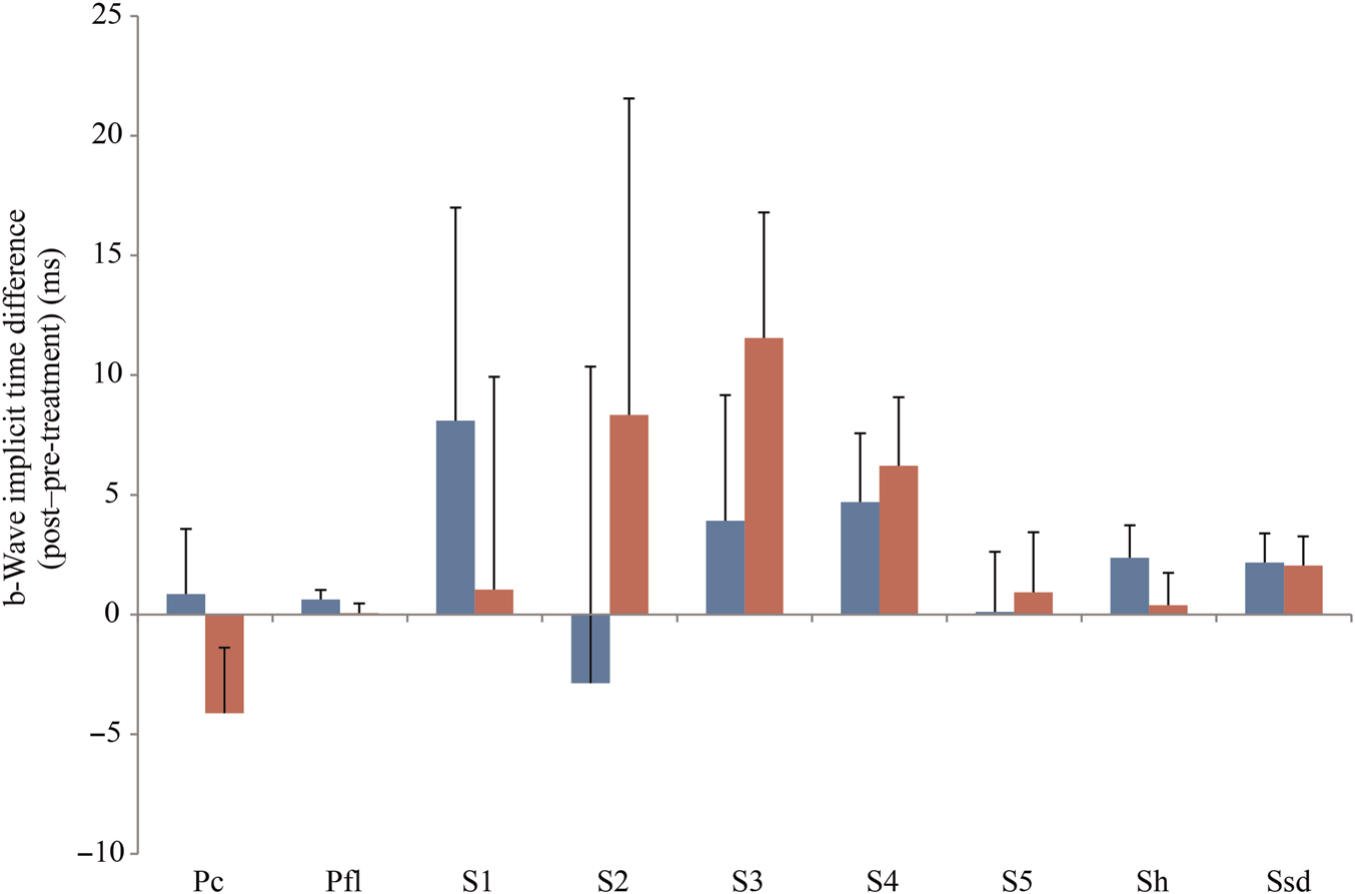
b-Wave implicit time difference from post- to pre-treatment tests in treatment (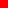) *v.* control (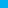) groups. Ssd and Sh: with higher light intensity stimulation (Ssd and Sh: 3 and 10 cd·s/m^2^, respectively) in the dark-adapted state, a- and b-wave responses are obtained, demonstrating mixed rod and cone function. Pc and Pfl: rods are desensitised using 30 cd·s/m^2^ of background light for 10 min, after which cones are stimulated using 3 cd·s/m^2^ in the light-adapted state. Pc and Pfl: single flash and 30 Hz flicker. S (S1–S5): using low intensity of light stimulation (0·01 cd·s/m^2^) in the dark-adapted state every 4 min up to 20 min of dark adaptation; using this low light intensity only the b-wave is obtained, corresponding to activity from the rod photoreceptors. Values are means, with standard errors represented by vertical bars. Ssd, electroretinography response from scotopic standard-intensity light stimulation; Sh, response from scotopic high-intensity light stimulation; Pc, photopic cone response; Pfl, photopic flicker (30 Hz) response.

Comparing changes from pre- to post-treatment levels, the dogs who received daily antioxidant supplementation demonstrated an increase in a-wave (Fig. 1) and b-wave amplitudes (Fig. 2), and a decrease in the implicit time for the a-wave (Fig. 3), and a decrease in implicit time for the b-wave (Fig. 4) when compared with those in the control group of dogs (*P* < 0·05).

All ERG a-wave amplitudes obtained increased in the treatment group compared with those of the dogs in the control group, with significant increase of amplitude in the scotopic high (*P* < 0·05) and photopic single flash cone responses (*P* < 0·05) (Fig. 1). The implicit time for a-waves under various testing conditions decreased, but this was only statistically significant for the photopic standard intensity (Fig. 3). For the b-wave amplitudes, all responses increased similarly, with significant increase in the scotopic high light intensity stimulation (*P* < 0·05), and significant increase in the responses of photopic single flash cone and 30 Hz flicker (*P* < 0·01 for both) recordings (Fig. 2). The implicit times for b-waves under various testing conditions showed one significant difference for a decreased implicit time with photopic standard intensity response (Fig. 4).

**Fig. 5.**
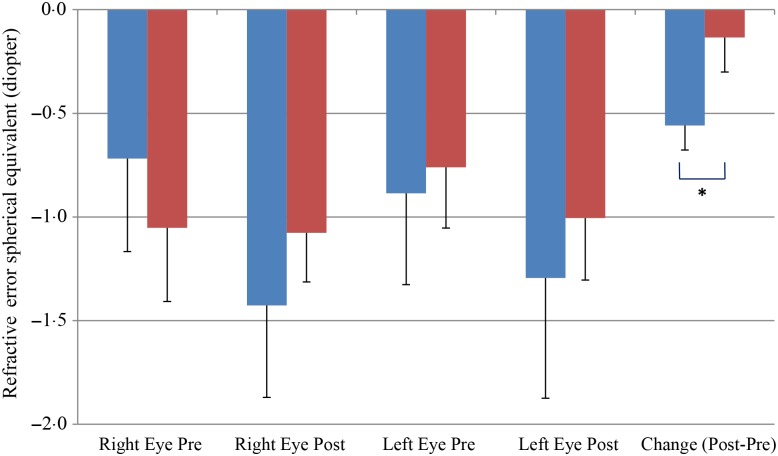
Change of refractive error from post- to pre-treatment tests in treatment (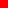) *v.* control (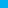) groups. Values are means, with standard errors represented by vertical bars. Significant treatment × time interaction: * *P* < 0·05.

We also observed a significant difference between the refractive error changes between the two groups. With all eyes combined, the control group had a refractive error change (post-treatment–pre-treatment) of −0·56 over the 6-month study period while the treatment group had a change of −0·13 (*P* < 0·05) (Fig. 5).

## Discussion

Prior to this study, there was one dog study showing that antioxidants including lutein and β-carotene decreased oxidative stress in sled dogs^(^^23^^)^, and there have been no published studies that have documented an effect from antioxidants such as carotenoids supplementation on retinal or visual function in dogs. We have shown that antioxidants can have significant effects on retinal function, as measured by ERG, and on delaying visual impairment as measured by auto-refractor.

There was a statistically significant difference observed between the treatment group and the control group in the photopic ERG parameters (Pc). In human studies, supplementation with xanthophylls such as lutein and zeaxanthin, as used in the present trial, have been associated with improvement of central cone vision^(^^20^^,^^21^^)^. The improvement of human light-adapted vision after supplementation suggests that supplemented dogs could potentially also benefit from these nutrients by improving retinal function.

These ERG changes are also in line with previous observations of fish oil supplementation that showed improvement of retinal function in young puppies fed high-dose *v*. low-dose fish oil supplements^(^^13^^)^. Based on previously observed age-related reductions in retinal function of both Yorkshire terriers and miniature poodles with increasing age^(^^2^^)^, it appears that retinal functional decline measured by ERG truly reflects an age-related decline. When comparing the ERG responses from 3- to 5-year-old to 10- to 14-year-old dogs, b-wave reduction was more prominent in photopic (30 %) compared with scotopic (15 %) conditions. Yorkshire terriers seemed to have more reduction (21·9 %) than miniature poodles (14·4 %).

Our study shows that nutritional factors can play an important role in preventing such age-related decline in retinal function and may even preserve visual function. In the untreated dogs, retinal function declined within the 6 months of this study, as indicated by a decrease of −6·1 % in Sh, −12·1 % in Pc, and −22·3 % in Pfl. In comparison, the treatment group had an increase of 27·2 % in Ssd (*v*. no change in the control group), 22·7 % in Sh, 14·4 % in Pc, and 44·4 % in Pfl, indicating an improvement in retinal function in the dark and light conditions over this 6-month treatment period. These changes suggest that the antioxidants tested in the present study have a significant impact (*P* < 0·05) on retinal responses.

We are the first to show that antioxidant supplementation can slow down refractive error change in dogs. The refractor error change showed a significantly difference between the control and treatment groups over the 6-month antioxidant supplementation time period. It is well known that the age-related lens sclerotic changes are associated with increased lens cloudiness^(^^3^^)^, increased lens reflective dot sizes indicating light scattering and blurry vision^(^^3^^)^, and increased refractive error towards a myopic shift^(^^6^^,^^7^^)^. Myopic shift of dog vision could have a significant native impact on dogs’ activities and performances such as retrieving target objects^(^^24^^)^.

Humans affected with nuclear sclerosis/nuclear cataract report visual disturbances resulting from a myopic shift (from hardening of the lens nucleus), astigmatism, a shift in contrast sensitivity (especially with low-contrast objects), glare and visual acuity reduction^(^^5^^)^. A myopic shift in the lens has been reported in older dogs^(^^25^^)^, presumably from a change in refractive status of the sclerotic lens nucleus, and this shift probably affects visual acuity^(^^26^^)^.

Antioxidants were able not only to improve retinal responses as measured by ERG but also to slow down the refractive error myopic shifting during a 6-month antioxidant supplementation trial in dogs. The strong effect sizes reported (Table 3) for various ERG response parameters are more meaningful than the *P* values for confirming the clinical impact of the supplementation. These results confirmed our hypotheses that these antioxidants do benefit dogs’ eyes, and improve their retinal and visual functions.

Several limitations may exist in the present study: The retinal structure was not evaluated in this study (e.g. by histopathology and/or optical coherence tomography). There is a great variability in ERG responses and the standard error in this study is high. Due to technical issues, a-wave Pc amplitude and implicate time were missed in one dog and b-wave Pc amplitude was missed in another dog in the treatment group during the post-treatment ERG measurements. As a result, these three measures for the post-treatment treatment group were results for five dogs instead of six dogs shown in Table 3. However, despite the large variation, several of the ERG recordings and refractive error changes showed statistically significant differences between treatment groups. An increased number of subjects in each group may further strengthen the differences found between treatment groups and give less variation.

As to the study diets, the control diet met the AAFCO (Association of American Feed Control Officials) requirements for vitamins C and E. The control diet contained no vitamin C and 39 mg/kg vitamin E. A slight decrease in b-wave amplitude was noticed in the control group over the 6-month study period, which might be attributed in part to the reduced vitamin C and E levels in the control diet. The antioxidant supplementation demonstrated a significant increase in retinal responses, a benefit beyond a typical commercial dog food containing vitamin C and E levels meeting AAFCO requirements. Whether a specific premium commercial dog food may benefit from such supplementation as in this study depends on the composition of the diet such as the levels of antioxidants and the bioavailability of the antioxidants in the specific diet. In addition, a synergic effect of the combined antioxidants is more likely with the supplementation as reported in this study rather than effects from individual antioxidants.

Since the study was done in healthy middle-aged dogs, the potential benefit of these antioxidant supplements cannot be extrapolated to dogs with retinal diseases, i.e. PRCD dogs. Further studies are needed to evaluate whether these supplements may benefit diseased retinas.

In summary, this study showed that antioxidants not only increased retinal function measured by scotopic and photopic ERG recordings, but also decreased the refractive error change in dogs fed the antioxidant supplements compared with dogs fed the control diets. This suggests that even in healthy dogs with normal eyes, a better retinal response can be obtained with antioxidant supplementation. Dogs, much like humans, experience retinal functional and visual functional decline with age. Antioxidant supplementation may be beneficial and effective in the long-term preservation and improvement of retinal function and the slowing of refractive error changes associated with ageing in dogs.
